# Iron responsive elements mRNA regulate Alzheimer’s amyloid precursor protein translation through iron sensing

**DOI:** 10.3389/fnagi.2025.1483913

**Published:** 2025-05-14

**Authors:** Mateen A. Khan

**Affiliations:** Department of Life Science, College of Science and General Studies, Alfaisal University, Riyadh, Saudi Arabia

**Keywords:** iron homeostasis, Alzheimer’s disease, iron responsive element, iron regulatory protein, amyloid precursor protein, IRE/IRP interactions

## Abstract

Iron responsive element (IREs) mRNA and iron regulatory proteins (IRPs) regulate iron homeostasis. 5′-untranslated region motifs of APP IREs fold into RNA stem loops bind to IRP to control translation. Through the 5’-UTR APP IREs, iron overload accelerated the translation of the Alzheimer’s amyloid precursor protein (APP). The protein synthesis activator eIF4F and the protein synthesis repressor IRP1 are the two types of proteins that IREs bind. Iron regulates the competitive binding of eIF4F and IRP1 to IRE. Iron causes the IRE and eIF4F to associate with one other, causing the dissociation of IRPs and altered translation. In order to control IRE-modulated expression of APP, messenger RNAs are becoming attractive targets for the development of small molecule therapeutics. Many mRNA interference strategies target the 2-D RNA structure, but messenger RNAs like rRNAs and tRNAs can fold into complicated, three-dimensional structures that add another level of complexity. IREs family is one of the few known 3-D mRNA regulatory elements. In this review, I present IREs structural and functional characteristics. For iron metabolism, the mRNAs encoding the proteins are controlled by this family of similar base sequences. Iron has a similar way of controlling the expression of Alzheimer’s APP as ferritin IRE RNA in their 5ÚTR. Further, iron mis regulation by IRPs can be investigated and contrasted using measurements of expression levels of APP, amyloid-*β* and tau formation. Accordingly, IRE-modulated APP expression in Alzheimer’s disease has great therapeutic potential through targeting mRNA structures.

## Introduction

Alzheimer’s is a central nervous system neurodegenerative disease that primarily affects the elderly and causes dementia (Santoro et al., 2025; Zheng and Wang, 2025). The development of extracellular *β*-amyloid (Aβ) peptide aggregation, intracellular hyperphosphorylated tau protein buildup in neurofibrillary formations, and an increased brain iron content are the hallmarks of Alzheimer’s disease (AD) (Olufunmilayo and Holsinger, 2023). Because iron is involved in many physiological functions of the brain including respiration, oxygen transport, DNA synthesis, energy metabolism, neuronal myelination, and neurotransmitter synthesis, it is essential to maintaining the normal physiological level of iron in the brain (Hare et al., 2013). Labile iron (Fe) catalyzes the creation of harmful reactive oxygen species that damage proteins, lipids, and DNA, maintaining cellular free iron pool is strictly regulated (Dusek et al., 2022). However, a number of neurodegenerative conditions, including Alzheimer’s, Parkinson’s (PD) illnesses, Friedreich ataxia (Fleming and Ponka, 2012), and other Central Nervous System (CNS) disorders, are linked to mismanagement of iron in the brain (Ward et al., 2014).

It has been documented that iron homeostasis and AD pathogenesis are directly related (Dusek et al., 2022). According to several studies (Ndayisaba et al., 2019; Martins et al., 2022), one of the primary causes of neuronal death in a variety of neurodegenerative illnesses is a mis-regulation in brain iron metabolism. The expression of APP mRNA in astrocytes and neuroblastoma cells (Long et al., 2022b) is influenced by iron levels in the brain. It has been shown that anomalies in iron metabolism, primarily in neurons or astrocytes, are the direct cause of neuroferritinopathy and ceruloplasminemia, which are generated by mutations in ferritin and ceruloplasminemia, respectively (Rouault, 2013). Relationship between the mutations in several genes, such as PANK2, ceruloplasmin, and ferritin light chain (FRT-L) (Baringer et al., 2023). Numerous genes involved in cellular iron metabolism, amyloid overexpression, and neuronal cell death have been linked to recent findings (Zhang et al., 2021; Dixon et al., 2012). These results point to a clear connection between brain iron mismanagement and amyloid accumulation in Alzheimer’s disease. Iron neurochemistry and amyloid *β* protein, which is associated with Alzheimer’s disease, have been related (Hare et al., 2015). Iron serves a comparable role in the translation of ferritin mRNA, which binds to iron responsive elements (IREs) in the 5’-UTRs of ferritin transcript, in the regulation of Alzheimer’s amyloid protein production. Iron is known to play a major role in the neurotoxicity associated with AD and PD, as evidenced by the presence of 5’UTR IRE sequences in the transcripts of the amyloid precursor protein and *α*-synuclein, the activity of APP as a neuronal iron export ferroxidase, and the ability of iron to enhance the neurotoxicity of amyloid *β* (Bush, 2013). Additionally, iron chelators have been used in multiple clinical trials and have demonstrated their effectiveness against neurodegeneration in several animal models (Dusek et al., 2022). Iron regulatory protein (IRP) is the primary regulator of intracellular iron homeostasis. It selectively binds to the conserved IREs found in the UTRs of mRNAs to post-transcriptionally regulate several genes involved in iron homeostasis (Wilkinson and Pantopoulos, 2014; Zhang et al., 2014). An IRE stem-loop structure is present in the 5’-UTR of the APP transcript. The presence of an IRE stem loop in the APP transcript indicates that this protein plays a function in iron homeostasis, as has been shown by other iron-regulated proteins. A functional IRE RNA stem-loop that is encoded by the APP 5’-UTR is a potential candidate for influencing APP production. The inability to control iron binding has an impact on how iron-regulatory proteins interact with IREs, which can have an impact on Alzheimer’s amyloid overexpression.

Intracellular iron homeostasis is regulated post transcriptionally by the IREs. When it comes to the mis-regulation of these important proteins during Alzheimer’s disease, the IRE RNA stem-loop structure may play a crucial role. The disruption of iron metabolism, ferritin RNA translation, and transferrin receptor (TfR) transcript stability in the gastrointestinal mucosa and central nervous system are all associated with the lack of IRP2. The APP 5’-UTR was impacted by intracellular iron levels in a manner that is consistent with iron-dependent control of intracellular APP production (Zhou and Tan, 2017; Bandyopadhyay and Rogers, 2014). IRPs are RNA-interacting proteins; their interaction with IRE regulates the translation of ferritin mRNA and the stability of TfR mRNA. Iron metabolism is regulated by the interaction of IRPs with IREs 5’-UTR mRNA transcripts (Rogers and Cahill, 2020).

IREs are present in the target mRNA’s 3′- and 5’-UTRs (Piccinelli and Samuelsson, 2007). IREs fold into bent A-RNA helices with terminal loops and form stem-loop structures made up of a 30-nt RNA helix (Chen and Olsthoorn, 2019). IRE RNA structures strongly bind to IRPs protein. By altering the distribution of iron metabolic mRNAs among complexes containing boosting eIFs and inhibitory IRPs, IRE-RNAs regulate the rates at which proteins are synthesized. The 146-nt 5’-UTR of the APP IRE stem-loop mRNA contains the CAGA box, which interacts to the IRP1 (Cho et al., 2010). The structure of the IRP is also altered by the binding of the APP IRE RNA. Within the IRE family, IRP binding stabilities differ by a factor of ten, representing the paired and unpaired bases of each IRE RNA. The graded iron responses *in vivo* are influenced by this variance (Cho et al., 2010). The development of the IRP/IRE complex is stabilized or destabilized by the amounts of cellular iron. The abnormalities in the IRP/IRE signaling pathway caused by iron overload in the brain tissues are responsible for the overexpression of amyloid, its aggregation, the loss of neurons, and the advancement of Alzheimer’s disease.

Cellular iron level controls both the APP transcript and the toxicity of amyloid. By affecting the mRNA translation and turnover of the APP, ferritin, transferrin receptor, and other iron-associated proteins, IRPs regulate the steady state of iron (Bandyopadhyay et al., 2013). Variations in the amounts of iron inside cells can impact the attachment of IRP1 to the IRE stem-loop, hence impacting the homoeostasis of APP, transferrin, and ferritin (Wang and Pantopoulos, 2012). IRPs could bind tightly to IREs found in the 3’-UTR of the TfR1 mRNA at low iron levels. This stabilizes the transcripts and raises the TfR1 protein level, which facilitates iron uptake. On the other hand, low iron levels also make it easier for IRPs to connect with IREs found in the ferroportin-1 mRNA and lower the ferroportin protein level, which exports iron. Consequently, the IRE-IRP connection for iron sensing raises the availability and uptake of iron in cells. Conversely, elevated iron levels diminish the affinity of IRPs to bind IRE, leading to a decrease in TfR1 mRNA stability and an increase in ferroportin mRNA translation, which work in concert to deplete cellular iron (Rogers and Cahill, 2020). So, understanding IRE binding affinity is crucial to understanding IRP-mediated iron homeostasis (Zhang et al., 2014).

Neuronal metal homeostasis is impacted by amyloid proteins, and metal ions are prevalent in synapse. According to reports, neurodegenerative illness may worsen if the metal steady state in neurons is disturbed (Adlard and Bush, 2018; Jiang et al., 2019). Micronutrients like iron (Fe), zinc (Zn), and copper (Cu) are essential for several brain activities, such as signal transmission, neurotransmitter synthesis, neuronal myelination, and defense against reactive oxygen species. Learning and memory impairments are brought on by a lack of these neuro-metals, which disrupt brain function. In the brain, iron is the neuro-metal that is most prevalent. Iron is a necessary cofactor for several cellular functions (Thirupathi and Chang, 2019). By attaching to the iron transporter ferroportin, APP controls the amount of iron that leaves cells (Wong et al., 2014). Moreover, iron controls the production of proteins bearing the iron responsive element sequence by binding to iron regulatory proteins (Zhou and Tan, 2017). The APP transcript’s IRE domain bears similarities to ferritin and other iron-binding proteins (Rogers et al., 2002). Therefore, APP can regulate the amounts of metal ions in neurons, which in turn controls the production of APP. As evidenced by the interactions between cytosolic aconitase and IRE-binding protein, IRP must undergo a critical conformational change to bind IRE RNA. This entails significant domain movements and conformational changes to the RNA-binding pocket process. These alterations in IRP1 structural conformation were detected by X-ray crystallography and circular dichroism (CD) spectroscopy after binding to IRE (Selezneva et al., 2013). Comparable techniques were used to clarify structural alterations that occur when eukaryotic translation initiation factors (eIFs) attach to the mRNA cap moiety and IRE RNA (Volpon et al., 2006; Khan et al., 2020). In this review, I aim to summarize what is currently known about the relationship between iron homeostasis and the IRE in the 5’UTR of APP mRNA, as well as how these molecules may be used for therapeutic and clinical purposes in Alzheimer’s disease. In addition, I examined the set of RNA sequences that make up the IRE family and confirmed that several IREs have binding potentials to IRPs. We investigated the significance of the pseudotriloop conformation of APP and ferritin IRE for binding to IRPs by utilizing biophysical and computational techniques (Khan et al., 2020; Khan et al., 2017; Khan et al., 2023) and the yeast three-hybrid system (Cho et al., 2010), which has been widely used to assay protein-RNA interactions *in vitro* (Khan et al., 2009; Khan et al., 2024) and *in vivo* (Zhang et al., 2020).

## Iron responsive elements (IREs) structure and function

**Figure 1:**
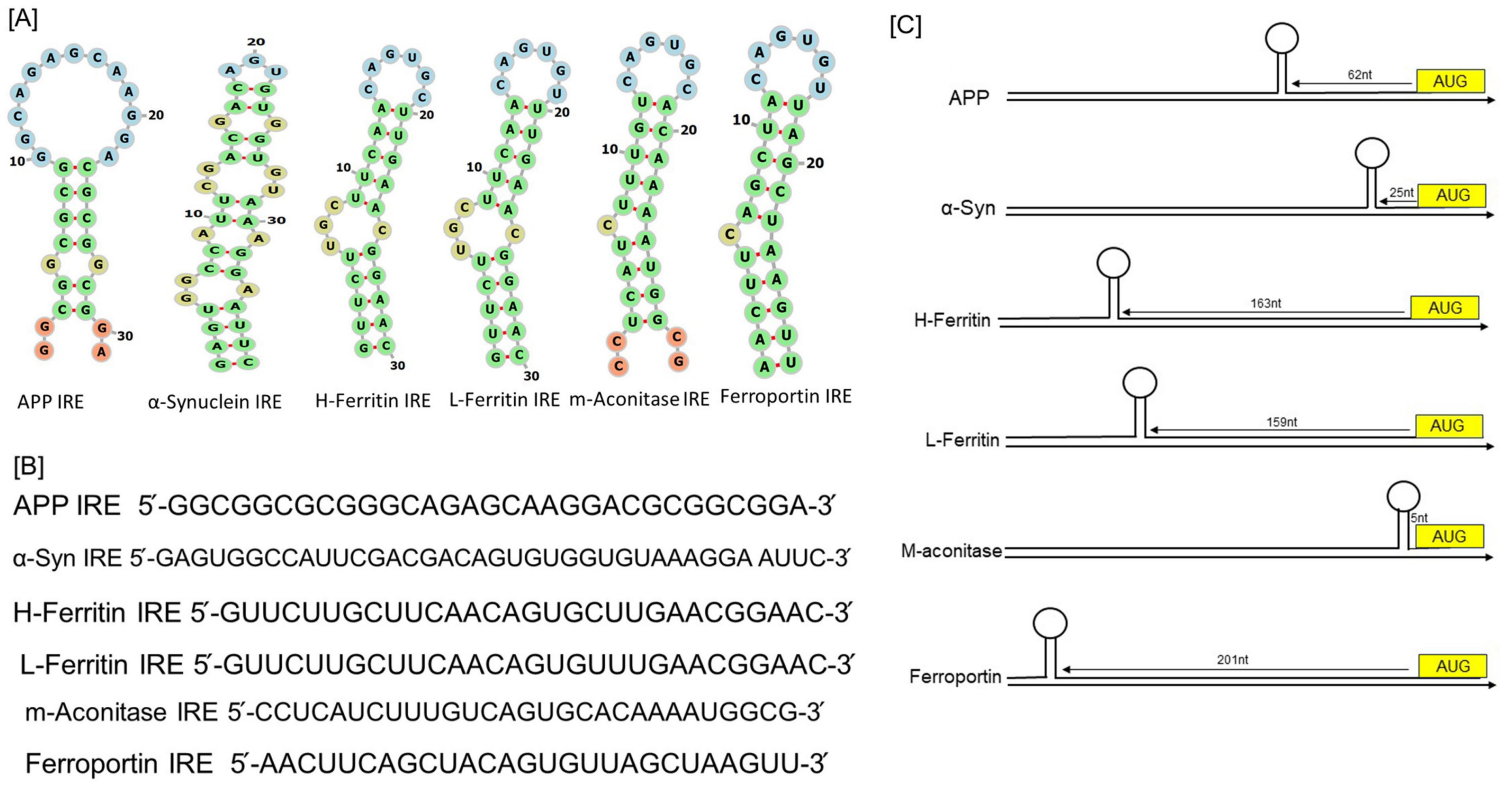
RNA stem-loops shared by the 5’-UTRs in the mRNAs of the neurodegenerative transcripts compared to iron regulatory proteins. **(A)** Potential iron regulating protein binding motifs and IRE-like AGU/AGA tri-loops have been identified in important transcripts associated with neurodegenerative diseases, including human APP, α-synuclein, H-ferritin, L-ferritin, mitochondrial aconitase, and ferroportin transcripts. **(B)** Sequences encoding the 5’-UTR specific IRE stem-loops in an APP and α-synuclein transcripts aligned with those of H-and L-ferritin, mitochondrial aconitase, and ferroportin. **(C)** Maps of the 5’-UTR IRE stem-loops in the transcripts of APP, α-synuclein, H- and L-ferritin, mitochondrial aconitase, and ferroportin.

A cis-acting messenger RNA regulatory motif with a distinct, highly conserved stem loop sequence and structure is known as an iron responsive element (IRE) (Volz, 2021). The foundation of iron-responsive elements is the RNA hairpins. The most prevalent RNA elements, hairpins, provide helpful handles for regulatory proteins to attach to Hentze et al. (2018) and Bevilacqua and Blose (2008). A conserved, six-nucleotide loop sits atop each IRE, which is around thirty nucleotides long and folded into a hairpin comprising two short helices divided by a bulge C (Figure 1). Mammals have a high degree of conservation in the structure and sequence of individual rings (IREs), which are typically composed of an apical loop motif and stem-loop element that are separated from a lower stem by a C-bulge (Leipuviene and Theil, 2007; Muckenthaler et al., 2008; Volz, 2008; Shen et al., 2023). The original IREs were discovered in ferritin mRNA. Numerous IRE-like structures have now been hypothesized in different mRNAs, some of which encode proteins not only important in iron homeostasis but also not exclusively (Rogers et al., 2011).

IRE-containing mRNAs have been found in a variety of other proteins recently, including *α*-synuclein in Parkinson disease (Wilkinson and Pantopoulos, 2014), α-hemoglobin stabilizing protein (dos Santos et al., 2008), and Alzheimer’s amyloid precursor protein (APP) (Cho et al., 2010; Rogers et al., 2002). The pseudotriloop is expected to be present in the secondary structure of APP IRE RNA. The G7 residue is projected to be a bulge base in the APP IRE RNA predicted structure, whereas the C8 residue is not (Cho et al., 2010). A functional IRE is also encoded in the 5’-UTR of the AD APP transcripts (Rogers et al., 2002). According to a reported (Rogers et al., 2002), the canonical loop motif of the H-ferritin IRE RNA stem loop and the APP IRE RNA are quite similar. Base pairing in the human APP IRE may produce a crucial AGA triloop (Cho et al., 2010). The RNA stem-loops encoded by the 5’-UTRs of transcripts associated with neurodegenerative diseases, namely those for *α*-synuclein and APP, are depicted in Figure 1A comparison to ferritin, the general IRE RNA. Every mRNA encodes distinct variants of an IRE RNA stem-loop that may attach to the 5’-UTRs of IRP translational repressors. Ferritin IRE/IRP1 complex (Khan et al., 2009) and the APP IRE/IRP1 complex (Khan et al., 2023) seem to have comparable binding stabilities. Two forms of conserved information are present in every member of the IREs family, information particular to the IRE RNA and information shared by all IREs. A short (9–10 bp), double-stranded helix with an unpaired C at the center that causes a bulge is present in all IRE RNA. Since all IRE RNAs create the RNA A-helix with the identical bulge C and terminal loop sequence, the changes in IRE sequences across various mRNAs are quite minimal. A conserved C-G base pair across the IRE terminal loop in all IRE RNA results in a bulge, an AGU, and a triloop (Figure 1A; Walden et al., 2006). A UGC/C or C bulge located five bases upstream of the terminal loop, which splits the hairpin stem into an upper and a lower stem, is present in IREs along with a highly conserved terminal loop. Five bases upstream of the terminal loop is a conserved UGC/C or C bulge that splits the hairpin stem into an upper stem and a lower stem.

In comparison to ferritin IRE (Zhou and Tan, 2017), mitochondrial aconitase IRE (Khan et al., 2009), and ferroportin IRE (Bandyopadhyay et al., 2006), Figure 1B illustrates the alignment of RNA bulges and base pair differences in RNA helix base pairs across members of the IRE RNA family of the 5’-UTRs in the neurodegenerative transcripts for APP IRE and *α*-synuclein IRE. This homology includes the ferritin 5’-UTR, which differs from the established APP IRE but yet encodes a sequence resembling the IRE (Khan, 2025). A 56% similarity between the ferritin and APP IRE RNA sequences in this region of the 5’-UTR was revealed by alignments. The predicted IRP1 binding AGU/AGA tri-loops, which have been demonstrated to be essential for IRP1 and IRP2 binding as well as translation suppression, and the CAGUGN loop domain of the canonical ferritin IRE are the focal points of this homology (Cho et al., 2010; Goforth et al., 2010). Varying mRNA stabilities (Khan et al., 2017; Khan et al., 2009; Theil and Goss, 2009) are a contributing factor to variations in the amounts of proteins encoded in IRE mRNAs within cells. The RNA sequence conservation of IRE RNAs encoding distinct proteins is lower (~80%) than the phylogenic conservation of a single IRE RNA (90%), which is required to achieve the current array of IRP binding constants. The 100% conservation of sequence homology between the mouse, rhesus monkey, and human APP IREs. The ferritin IRE RNA sequences of vertebrates, including humans, chickens, frogs, and mice, differ considerably (Piccinelli and Samuelsson, 2007; Cho et al., 2010).

Figure 1C shows how the IRE-like RNA stem loop locations are arranged in the 5’-UTRs of the transcripts for human APP, α-synuclein, H-ferritin, L-ferritin, mitochondrial-aconitase, and ferroportin. It is crucial to locate the IRE inside the mRNA UTRs. The regulatory efficiency of 5’-UTRs can be influenced by the distance of the IRE from the mRNA cap and AUG start site (Zhou and Tan, 2017). Depending on the gene type, these IRE to start and IRE to cap distances can change (Figure 1C). The conservation of an individual IRE RNA, like APP mRNA, is identical across species; ferritin mRNA, on the other hand, differs significantly less from IRE RNA sequence variations in other mRNAs belonging to the same species (Piccinelli and Samuelsson, 2007). The stem of the lengthier IRE RNA sequences often contains more base pairs below the bulged C. All of the IRE RNAs in the 5’UTR boost translation when iron is low, restrict ribosome binding, and increase translation when iron is high; however, the IRE RNA sequences that have a higher affinity for regulatory proteins respond to iron more quantitatively. We identified the existence of a completely functional iron responsive element stem loop in the APP 5’UTR, similar to ferritin (Khan et al., 2023). The sequence alignment between the known IRE in ferritin mRNA and the 5’UTR sequences of APP RNA is displayed in Figure 1. The APP 5’UTR sequences and the IRE in H-ferritin mRNA showed an overall 67% sequence similarity according to these alignments.

Multiple independent transfection studies evaluated the entire functionality of this unique iron sensitive region in the 5’UTR of APP mRNA (Rogers et al., 2002). We have recently (Khan et al., 2023), predicted the APP IRE RNA secondary and tertiary structures, and docking models of APP IRE RNA/IRP1 complex. Ferritin IRE RNA/IRP1 complex was predicted before (Walden et al., 2006). The folded RNA secondary structure predictions were based on the APP IRE RNA sequence. Figure 1 illustrates the projected APP RNA stem loop, which has a Gibbs free energy value of −10 kcal/mol. Thermodynamic studies are used to predict the secondary structure of APP IRE mRNA, which is primarily composed of base-paired stems and hairpin loops (Khan et al., 2023). Similarities exist between the ferritin IRE mRNA stem-loop structure and the secondary structure of APP IRE RNA.

The IRE RNA family exhibits quantitative differences in its interaction with iron signal and other cellular macromolecules, including the IRP repressor, and translation initiation factors activators. The reason why the same amount of iron in the same tissue, like the liver, enhanced ferritin protein biosynthesis more than m-aconitase biosynthesis *in vivo* can be attributed to the slight structural variations in IRE RNAs. More amyloid protein biosynthesis was observed in brain tissue compared to mitochondrial ferritin and aconitase protein synthesis. A greater proportion of APP mRNA molecules than ferritin and ferritin mRNA molecules are bound to IRP when cellular iron levels are low. Comparing ferritin and mitochondrial aconitase mRNA, APP IRE RNA/protein dissociation is smaller (Khan et al., 2023; Khan et al., 2009; Khan et al., 2014), which may be due to differences in the way the various encoded proteins are used metabolically. The amount of APP IRE RNA molecules that become available for initiation factor and ribosome binding is disproportionately bigger than for ferritin and m-aconitase mRNAs when IRE RNA/IRP binding is impaired by elevated iron levels, which results in the expression of more APP protein in the brain. It was demonstrated by the RNA electrophoretic mobility shift experiment that IRP1 binds to the APP IRE stem loop specifically (Cho et al., 2010). RNA gel shift tests, on the other hand, showed that the mutant APP 5’UTR cRNA probe no longer binds to IRP (Rogers et al., 2002). IRP plays a crucial role in regulating the iron-dependent translation of APP expression in brain cells by preferentially interacting with the APP 5’UTR. The high binding of an IRP to the APP 5’UTR implies that the APP protein plays a crucial role in iron metabolism. If IRP expression can be functionally related to APP expression, comparing the relative affinity measurement of IRP as binding partners to the IRE 5’UTR will provide valuable information for screening novel lead compounds that limit APP translation.

IRP is the regulatory protein that can identify all IRE RNA configurations (Khan et al., 2009; Goforth et al., 2010). A series of RNA-protein complexes is produced by subtle, conserved variations in the IRE structure and sequence. The RNA-protein complex binding stability varies slightly throughout members of the IRE RNA family. There are physiological implications to the changes in IRE RNA-IRP stability. For instance, ferritin and m-aconitase IRE RNA/IRP have dissociation constants of 14.2 nM and 129 nM under the same conditions (Khan et al., 2009), whereas the dissociation constant for APP RNA/IRP binding in a solution is 32 nM (Khan et al., 2023). The 30-nucleotide IRE RNA sequences (Khan et al., 2017; Khan et al., 2023; Khan et al., 2009) that are embedded in mRNAs that are hundreds of nucleotides long were the RNA targets. While some of these RNA elements were predicted by computational analysis, such as the IRE structure found in *α*-synuclein mRNA 5’UTR, have not been reported for direct binding to IRPs, their involvement in transcriptional regulation has been reported (Chen and Olsthoorn, 2019). Some of these RNA elements were discovered by immunoprecipitation and were shown to have IRP binding affinity *in vitro* using electrophoretic mobility shift assays (Wilkinson and Pantopoulos, 2014; Cho et al., 2010). The fraction of IRE RNA inactivated by IRP binding will vary for each IRE RNA at any given moment due to quantitatively variable IRP binding affinities for each distinct IRE RNA (Khan, 2024). Compared to APP IRE RNA, ferritin IRE RNA forms a far more stable complex with IRP. Due to this, the translation of ferritin mRNA is more robust to slight variations in intracellular iron concentration than that of the mitochondrial-aconitase RNA and amyloid precursor protein, which together form the less stable IRE RNA/IRP complex. The structural differences between the APP IRE RNA and the ferritin IRE RNA correspond to functional differences in cell metabolism of each protein encoded in an IRE RNA, as each requires ferritin for iron concentration activities continuously, while the APP requires it on a periodic basis.

## Functional interaction of IRP/IRE stem loop in translation

**Figure 2:**
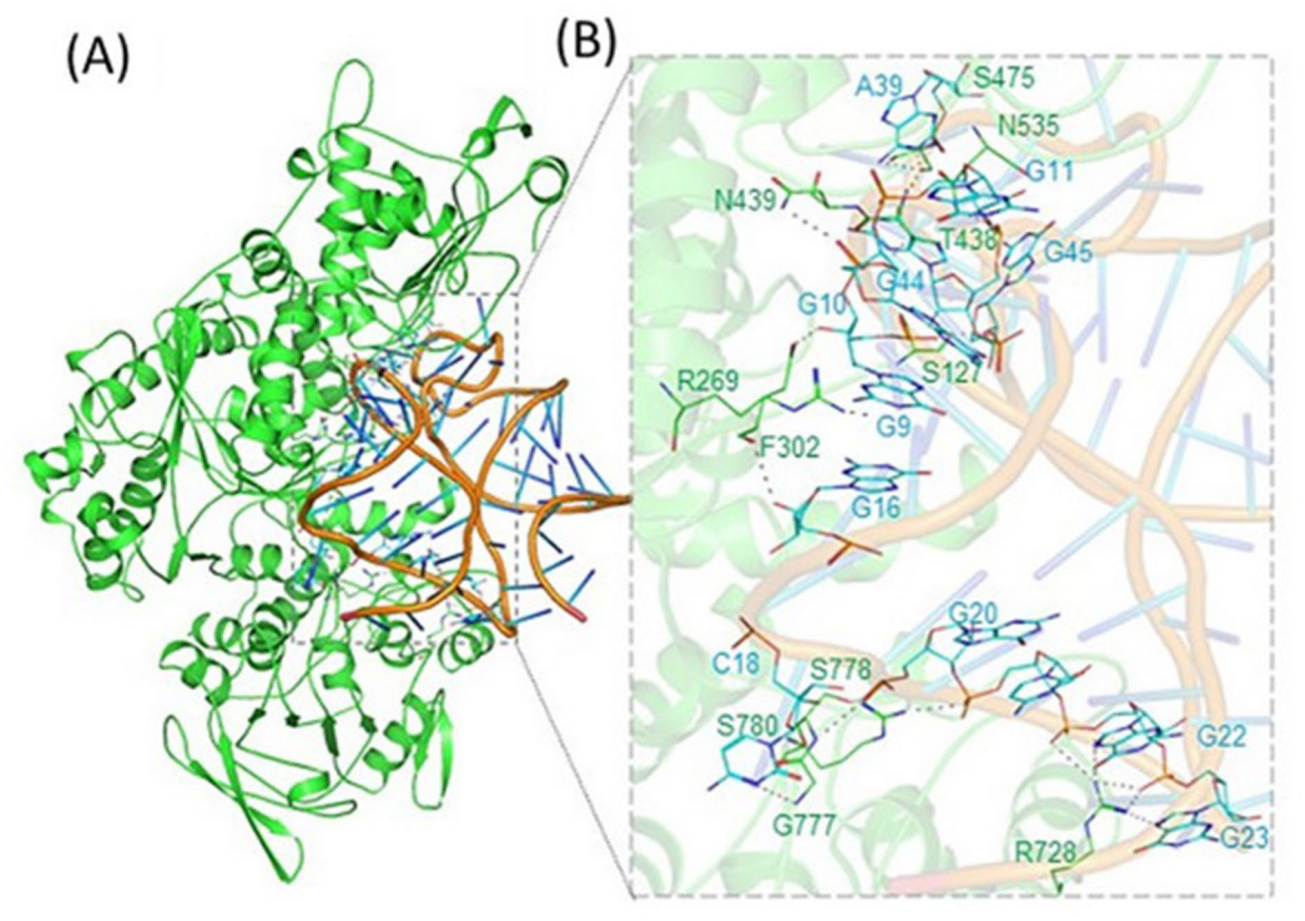
Binding of APP IRE RNA to IRP1. IRP1 was bound in a cleft by APP IRE RNA. **(A)** Ribbon diagram of IRP1 with APP IRE. **(B)** Zoomed ribbon diagram of IRP1 displaying interaction with APP IRE. Amino acid residues are in direct contact with IRE RNA in the APP IRE RNA/ IRP1 complex. Figure modified from the original publication in Khan et al. (2023).

**Figure 3:**
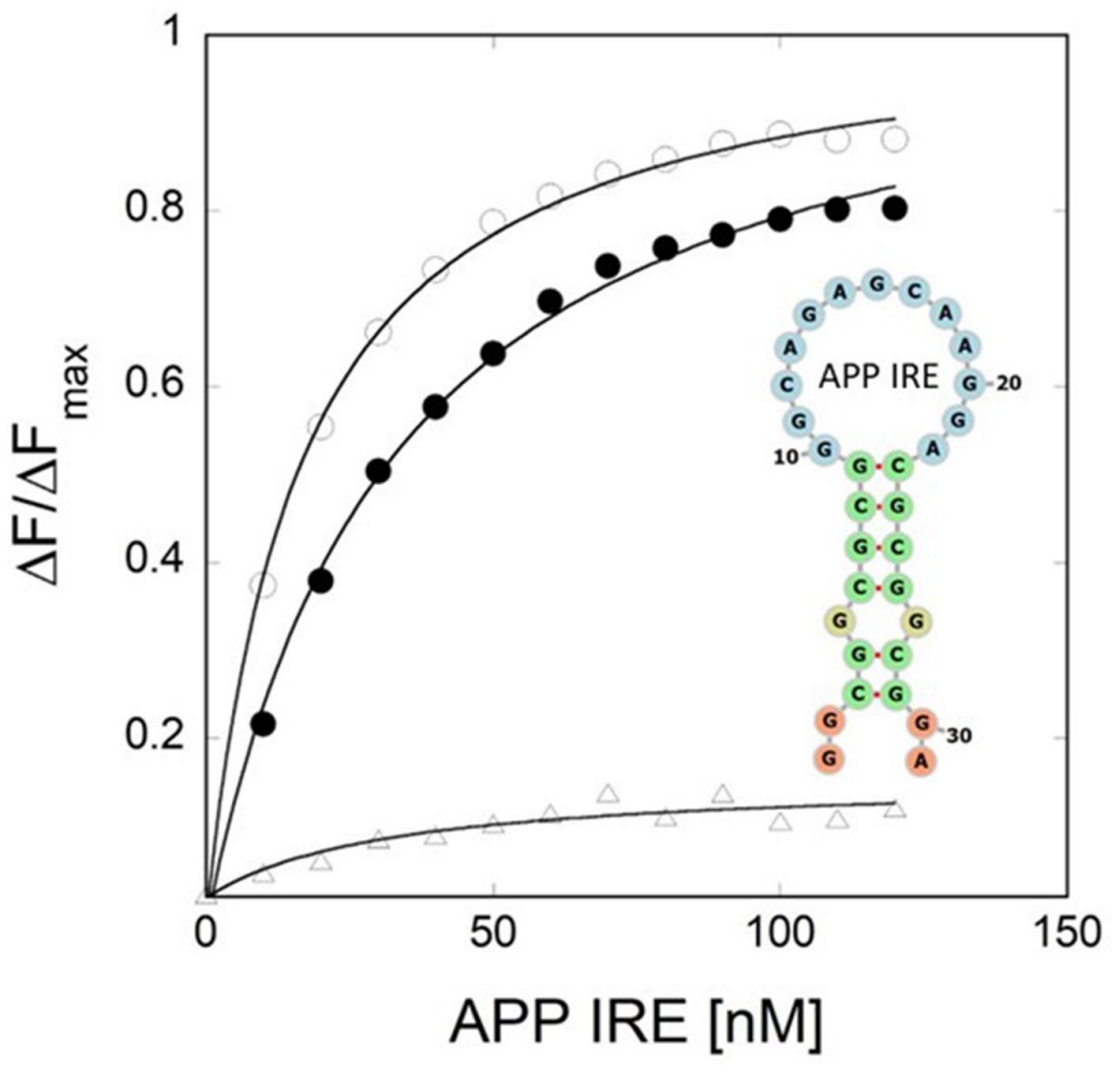
Iron weakens APP IRE RNA/IRP1 interaction. Khan et al. (2023) provided the binding curve and conserved IRE secondary structure.

The IRPs (IRP1/IRP2) proteins are recognized by the highly specific element known as the IRE. IRPs recognize IREs in a very specific way, allowing them to control the fate of mRNAs containing IREs by either stabilizing or preventing their translation (Muckenthaler et al., 2008; Maio et al., 2021). The translation of ferritin is induced by the iron-dependent interaction of IRPs with IRE stem loops at the 5’UTR, which increases the cell’s ability to store iron and preserves the survival of neurons under oxidative stress (Cho et al., 2010; Venkataramani et al., 2018; Hentze et al., 2010). Increased or decreased protein expression results from the association of IRE/IRP1 complexes, contingent on whether the IRE is in the 3′ or 5’ UTR. By regulating the transferrin receptor’s mRNA stability through 3’UTR specific RNA protein interactions, IRPs regulate the rates at which cells acquire iron (Rogers and Cahill, 2020; Rogers et al., 2008; Sanchez et al., 2011). The canonical interaction that regulates iron homeostasis in cells is the one that occurs between iron regulatory proteins and iron responsive element RNA stem loops (IRP/IRE interaction) (Figures 2, 3; Khan et al., 2023). The C bulge and the AGU apical loop are where the IRP binds to IREs primarily. Maintaining the orientation of the loop and the bulge for appropriate IRE/IRP binding is a crucial function of both the lower stem of the IRE, located below the bulge, and the upper stem of the IRE, situated between the terminal loop and the bulge. The structural conformation and functional ability of RNA to bind to a protein molecule determine its function (Kim and Gu, 2014).

**Figure 4:**
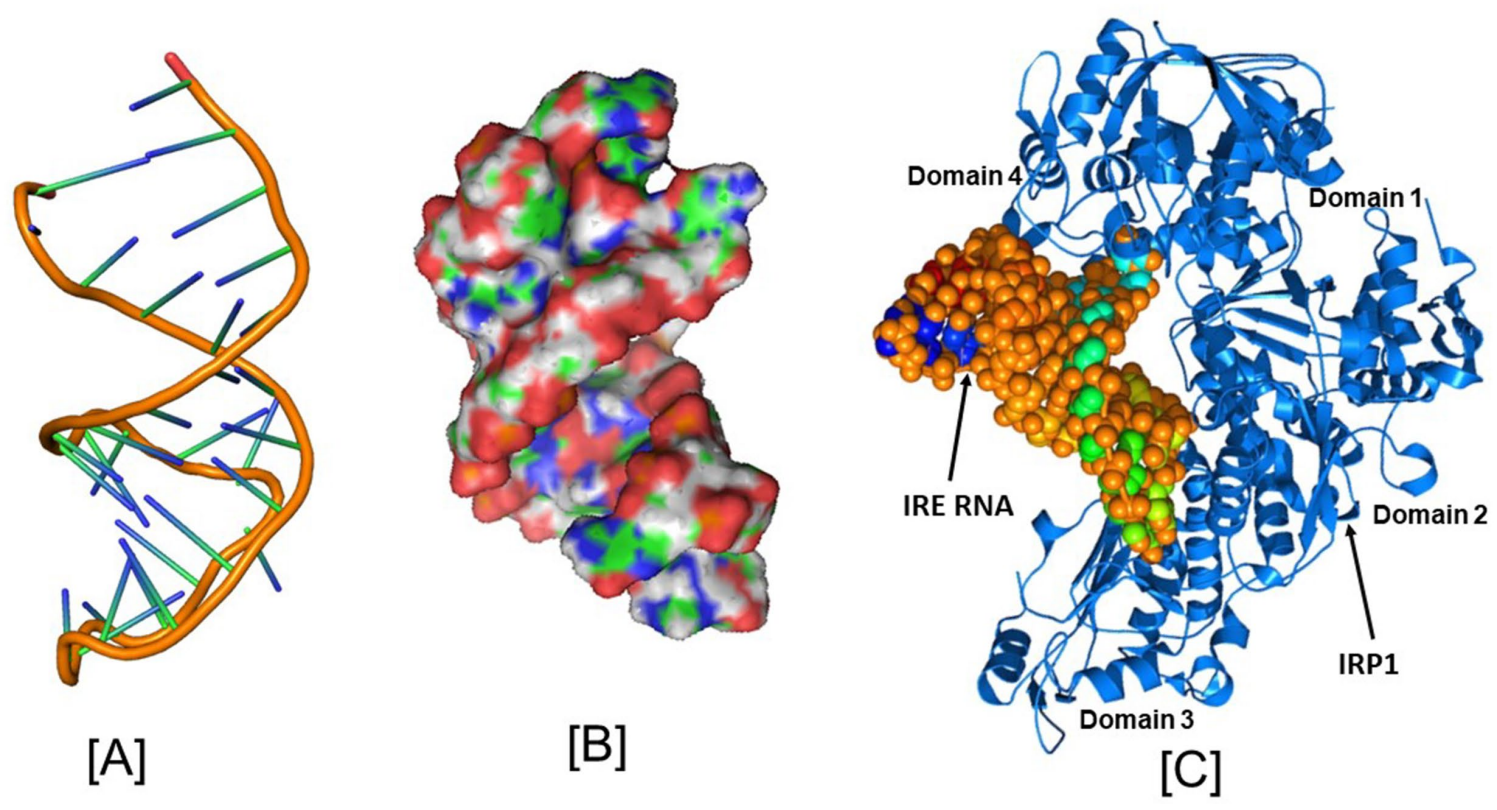
Structural models of the iron responsive elements and its complex with IRP1. **(A)** Secondary, **(B)** Tertiary structural models of the APP Iron Responsive Element (IRE) RNA, and **(C)** Iron Regulatory Protein (IRP1) structural model in conjunction with IRE RNA. PyMOL and PDB files were used in the creation of the images. Figures that were initially published in Khan et al. (2023) and Khan et al. (2014) have been modified.

To identify the nucleotide residues that oversee the IRE RNA conformation and the RNA/protein interaction, it is crucial to predict the tertiary structure. Like ferritin, the 5’UTR of APP mRNA regulates APP production through the interaction of APP IRE/IRP at the translational stage in response to iron (Rogers and Cahill, 2020). The APP IRE RNA structural model and a typical model of the IRE RNA/IRP1 complex are shown in Figures 2, 4A–C (Khan et al., 2023; Khan et al., 2014). The precise binding mode of the IRE RNA/IRP1 interaction study of the RNA in the IRP1 protein’s binding pocket is explored in Figure 4C. The IRE RNA is bent, and the IRP1 protein has an L shape. Between IRP1 protein domains 1–2 and 4, IRE is introduced into the complex (Figure 4C). The IRE RNA/IRP1 complex only has two contact sites that are widely apart in order to establish binding selectivity (Walden et al., 2006; Walden et al., 2012). Bonds formed to the terminal loop and stem interrupting C through two distinct binding sites are the primary means by which IRE RNA interacts with IRP1 (Walden et al., 2012).

IRP1 was shown to interact with IRE largely via hydrogen bonding between the phosphate bonds of the AGU/AGA trinucleotide loop and amino acids in the cleft between domains 2 and 3 of IRP1 (Walden et al., 2006). The bulge C interacts with ten amino acids in domain 4 of IRP1. IRP1 selectively binds to APP mRNA and that this selective interaction takes place via the functional IRE sequences in the 5’-UTR of the transcript (Cho et al., 2010). The complex has a high affinity and specificity because of the about twenty RNA/protein linkages that are dispersed between the binding sites. Contacts A15, G16, and U17 are crucial for identification in the apical loop. At a location that is blocked by domains 1 and 2 in the globular form, ten connections are made between amino acids in a pocket created in domain 3 and A15 and G16 in the pseudo-tri-loop at the RNA terminus. In IRP1 domain 4, eight bonds are formed between C8 and amino acids. Between the amino acids in IRP1 domain 4 and the stem below C8, there are four more linkages (Walden et al., 2006). In the RNA/protein interaction, unpaired U19 in the hexa-loop is flipped out of the RNA helix, unpaired U6 is tucked into the minor groove, and the RNA backbone is twisted by a strong mid-helix turn in addition to flipping out of terminal loop bases A15 and G16 and helix bulge C8 (Selezneva et al., 2013).

Conformational modifications in the RNA and probably the unliganded protein are necessary to explain the discrepancies between the solution structures of free RNA and protein bound IRE RNA. A significant portion of the IRE RNA’s surface is open to interactions with other proteins, RNA, and metal ions in the RNA/protein crystal structure (Selezneva et al., 2006). It seems likely that IRP1 uses the same bonding strategy to bind all naturally occurring IRE RNA. The structure of the IRE RNA bound to IRP1 is different from the predominant structure of the RNA in solution, highlighting the significance of conformational flexibility for this high affinity binding and suggesting that the conformational flexibility of the IRE RNA may play a role in the affinity differences observed between these IRE RNA/IRP protein bindings. The majority of the IRE hydrogen bonds with IRP1 when an IRE has an AGU triplet at positions A15, G16, and U17 at the apex of the IRE motif. Naturally, hydrogen bonds influence specificity but do not explain binding strength (Volz, 2021). For appropriate IRE/IRP control, these interactions are required. There may be less IRE/IRP binding if this apical loop is not A15 G16 U17, however it might not provide *in vivo* regulatory function.

Like ferritin IRE RNA binding to IRP1, the Alzheimer’s amyloid precursor protein IRE RNA bound in the binding pocket of IRP1 (Figures 2, 4). With a set of the IRP1 binding site’s functionally active residues, APP IRE RNA bound IRP1 in a cleft. The APP IRE RNA and IRP1 complex are held together by many bonds. The RNA terminal and amino acid residues of IRP1 make many hydrogen bonds in a pocket created in domain 3 at a location that is blocked by domains 1 and 2 in the globular form (Figure 2B). During the complex formation process, the stem-loop of APP IRE RNA and the amino acid residues of IRP1 (Ser127, Arg269, Phe302, Thr438, Asn439, Ser475, Asn535, Arg728, Gly777, Ser778, and Ser780) engage in hydrogen bonding interactions (Khan et al., 2023). In domain 4 of IRP1, additional bonds develop between the amino acids and the IRE stem. The APP IRE RNA in the IRE RNA/IRP1 complex flips out of its terminal loop bases, and the IRE RNA backbone is deformed by an abrupt mid-helix turn. The attached APP IRE RNA is in intimate touch with these amino acid residues. IRP1/APP IRE RNA complex stability is provided by hydrogen bonds. The structural complementarity of APP IRE RNA allowed it to fit tightly into the IRP1 protein’s binding pocket (Khan et al., 2023). The biggest changes in IRP1 binding in solution are seen in the *in vivo* response to iron levels, where ferritin, mitochondrial aconitase IRE RNAs and Alzheimer’s amyloid precursor protein differ by at least an order of magnitude (Figure 3; Khan et al., 2023; Khan et al., 2009; Goforth et al., 2010).

APP, ferritin, and mitochondrial aconitase IRE RNA have varying relative binding affinities with IRP1, which vary by approximately ten times, as determined by either mobility shift in gel electrophoresis or fluorescence quenching in solution. For solution fluorescence, the nanomolar binding affinity (Khan et al., 2009) contrasts with the picomolar binding affinity (Cho et al., 2010) from gel shifts. One possibility for this discrepancy is an adsorptive ingredient in the gels. In comparison to APP and m-aconitase IRE RNA, ferritin IRE RNA binds IRP1 most firmly (Khan et al., 2023; Khan et al., 2009), which is indicative of faster helix bending kinetics during protein binding (Khan et al., 2017). Since the contact sites of IRE RNA in IRP1 have a conserved structure, variations in the stability of the IRE RNA/IRP1 complex must be caused by variations in helical structure. Increasing the length of the upper stem or rupturing the helices above and below the C bulge changes the binding affinity of IRP1. Quantitative variations in IRP1 binding affinity and the extent of the iron response *in vivo* are correlated with natural variations in helix base pairs of IRE carrying RNAs coding for distinct proteins.

The essential control of iron homeostasis at the cellular level is provided by the IRP/IRE RNA machinery, which post-transcriptionally modifies the expression of target genes in accordance with cellular iron status. The effects of iron concentrations are demonstrated by the addition of chelators, as demonstrated by multiple experiments involving the Alzheimer amyloid precursor protein (APP) IRE RNA. As seen for several IRE RNAs in a range of cultured cell types, cells treated to the iron chelator desferrioxamine enhanced binding of IRP1 to IRE RNA (Cho et al., 2010). Prior to the current findings that iron destabilized APP IRE RNA/IRP1 complexes (Figure 3), the molecular mechanism was unclear (Khan et al., 2023). The specific architectures of the IRE RNA/IRP1 sequences affect how much of an iron effect there is. The impact of nature’s manipulation of riboregulation among IRE RNAs is evident in the APP IRE RNA/IRP1 binding affinity, which dropped by 4-fold (Khan et al., 2023) while ferritin and mitochondrial-aconitase IRE RNA/IRP1 binding fell by 20- and 5-fold (Khan et al., 2009). Based on NMR spectra, ethidium bromide displacement, and the lack of projected metal ion binding sites on IRP beyond the [4Fe-4S] cluster insertion site, metal ions bind straight to the IRE RNA. Furthermore, ferritin IRE RNA binding by eIF4F is iron-sensitive. Ferritin IRE RNA’s binding affinity with eIF4F was enhanced by iron (Ma et al., 2012; Khan, 2020). Iron, therefore, drives the binding competition away from IRP and toward eIF4F as IRP and eIF4F fight with each other for IRE RNA binding. Ultimately, several RNA/protein bonds are involved in the complex formation process; these bonds increased with the addition of iron for IRE RNA/eIF4F complexes and reduced with IRE RNA/IRP1 complexes. The repressor protein IRP1 was released because of iron-induced IRE RNA conformational changes, and increased binding of the eukaryotic initiation factor eIF4F led to an increase in translation (Ma et al., 2012; Khan, 2022).

## Iron-dependent IRE mRNA translation regulation

The existence of an IRE in the 5’UTR of the amyloid precursor protein transcript has been used to establish a direct connection between iron homeostasis and the etiology of Alzheimer’s disease (Zhou and Tan, 2017; Rogers et al., 2002). In a manner that mirrors the iron-dependent control of intracellular APP production, APP 5’UTR is thus selectively responsive to intracellular iron levels. Iron levels have been demonstrated to influence the translation of APP protein mRNA in astrocytes and neuroblastoma cells by a mechanism like iron control of ferritin L and ferritin H mRNA translation through IREs in their 5’-UTRs (Rogers et al., 2002; Rogers et al., 2008). The signaling cascade of iron regulatory proteins (IRPs) and iron responsive elements (IREs) modifies the cellular iron balance at the translational level (Zhang et al., 2014). RNA binding proteins like IRPs are known to regulate translation through their inducible interactions with IRE. Iron can have opposing effects on distinct IRE RNAs due to the two distinct positions of IRE RNAs in mRNA when IREs are present in the 5’-UTR and control the binding of ribosomes. Initiation factor and ribosome binding are made possible by iron signals, which also promote mRNA translation. Conversely, when the IRE is present in the 3’-UTR, it controls target mRNA nuclease binding and mRNA breakdown. Rather than controlling nuclease binding, the majority of IREs that have been identified control ribosome binding. IRPs could function as both a translational enhancer and an inhibitor (Theil, 2015).

The body struggles to keep the balance of iron in its cells at the right level. The expression and control of iron regulatory, storage, transport, and carrier proteins determine the equilibrium of the cellular iron level. The identification of iron responsive elements (IREs) in the untranslated regions (UTRs) of the mRNAs encoding ferritin and transferrin receptor marked the beginning of the description of the IRE/IRP regulation mechanism. Target mRNA with IRE motifs in the 3’-UTR includes five copies of TfR and DMT-1, while transcripts with IRE motifs in the 5’-UTR include both subunits of ferritin H and ferritin L, ferroportin, and aminolevulinic acid synthetase (Kato et al., 2007).

**Figure 5:**
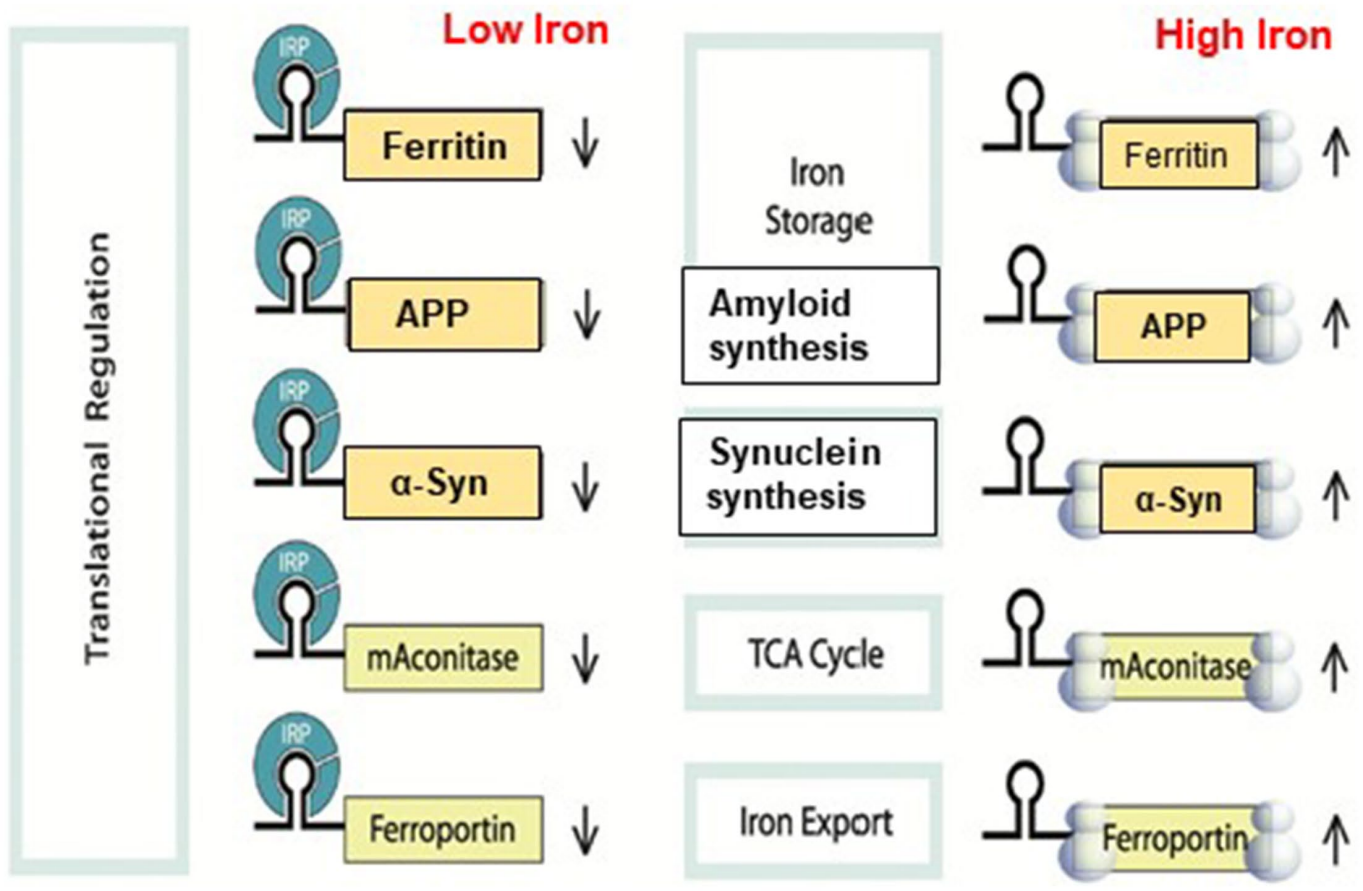
A model that illustrates how binding of IRE RNA to IRP causes iron to stimulate the translation of iron regulatory proteins. Protein expression and depression according to cellular iron levels (high or low). This figure was amended from Khan et al. (2009).

The IRP/IRE signaling system for translation control is shown in Figure 5. Although IRP binding to 5’-UTR is known to restrict mRNA translation and to limit ribosome binding, the specific stages involved in building the translation initiation factors complex that are hindered are still unknown. Target mRNA in iron-deficient cells can be shielded from endonuclease cleavage by IRP binding to an IRE at the 3’-UTR of transcripts. Thus, the interaction of IRPs with a 3’-UTR IRE can increase target mRNA translation and prolong the half-life of transcripts. On the other hand, target transcripts in iron-depleted cells are less likely to be translated due to endonuclease assault and degradation caused by IRP’s separation from an IRE at the 3’-UTR. When iron overload occurs, the IRP/IRE interaction can both destabilize TfR mRNA and disrupt and enhance ferritin transcript translation. Consequently, in conditions of iron overload, iron export and storage might be augmented while iron absorption is restricted (Hentze et al., 2010). It was not known until recently how cellular iron signals alter IRP’s affinity for APP IRE RNA (Khan et al., 2023).

Furthermore, the process of binding ribosomes is quite intricate and involves the binding of numerous initiation factors and proteins to form an initiation complex that includes ribosomal subunits, initiator tRNA, and mRNA. It is still unknown exactly what happens in what order IRP is released and an initiation complex with an IRE RNA is put together. Because there is little distance between the start of the mRNA and the IRE, several characteristics imply that IRE structures work in concert with other components of the mRNA structure. The initiator AUG is embedded in the IRE RNA of some IRE RNAs, such as m-aconitase; the functional significance of initiation at the IRE is unknown. To control the stability of a protein repressor complex that prevents ribosome binding and protein synthesis, these IREs structures bind iron preferentially. Binding the eIF4F protein synthesis initiation factor results in positive regulation. The common IRE RNA loop and bulge that comprise the distinct protein binding sites for each IRE are used to characterize the IRE RNA. Changes in the base pairs of the individual IRE RNA helix alter the binding of metabolites and repressor proteins (IRPs), converting protein production *in vivo* environmental iron. Ferritin protein synthesis consumes the iron signal, creating a regulatory feedback loop with iron. Consequently, ferritin protein reduces the amount of free cellular iron, raises the binding of IRP1 to ferritin IRE RNA, and lowers the rates of ferritin protein synthesis.

## Iron influence IRE stem loop binding to IRP1 and eIF4F

The iron regulatory protein’s ability to recognize IREs and control target mRNA translation preserves iron homeostasis. Iron has a direct role in the dissociation of the IRE/IRP1 complex because the IRE RNA binds metal ions, including physiologically relevant iron, to reduce IRE RNA/IRP stability (Khan et al., 2023; Khan et al., 2009; Ma et al., 2012). Figure 5 shows the detailed translation regulation processes of the IRP/IRE signaling system. IRPs could both promote and inhibit translation (Piccinelli and Samuelsson, 2007; Pantopoulos, 2004). These systems control iron homeostasis at several levels, resulting in fine-tuning of iron transport, storage, and regulation at the cellular and systemic levels *in vivo*. However, an imbalance in the iron regulating system results in an excess of iron in the brain’s tissues, which kills neurons (Ke and Ming, 2003). There have been reports of high levels of iron in the brain associated with several neurodegenerative diseases, including Alzheimer’s and Parkinson’s (Liu et al., 2022; Gao et al., 2025). Raising the quantity of iron within cells can modify not just the shape of IRE RNA, which in turn affects IRP and eIF4F binding, but also the IRP protein itself. The capacity of IRP1 to bind IRE RNA is lost when it binds an iron–sulfur cluster and transforms into cytosolic aconitase. Thus, IRP1 conversion to cytoplasmic aconitase increases and IRP1 availability to bind to IRE RNA declines as iron concentrations and iron–sulfur cluster formation increase (Netz et al., 2014). Therefore, lowering cellular iron concentrations reduces IRE RNA/IRP1 binding, increases IRP2 degradation, and modifies IRE RNA conformations to reduce IRP binding (Salahudeen et al., 2009; Deck et al., 2009). Iron excess can cause the IRP/IRE interaction to be disrupted, which can lead to the translation of ferritin and ferroportin transcripts and the destabilization of TfR and DMT1 mRNA (Pantopoulos, 2004; Pantopoulos, 2015; Pantopoulos et al., 2012). Therefore, in conditions of iron overload, iron export and storage can be increased while iron absorption is blocked (Hentze et al., 2010). Iron homeostasis will be hampered by disruption of the IRP/IRE signaling system, which helps to precipitate and worsen Alzheimer’s disease. We have disclosed a new mechanism for the iron-induced modification of target mRNA translation (Ma et al., 2012).

The binding of eukaryotic initiation factor (eIF4F) to the 5′ cap of mRNA initiates the process of protein synthesis. eIF4F is a supramolecular complex made up of the scaffolding protein eIF4G, cap binding protein eIF4E, RNA binding protein eIF4B and helicase eIF4A (Khan et al., 2020). This complex may also interact with IRP. Since eIF4F binds to IRE RNA competitively with IRP, showing that two proteins occupy the overlapping binding sites, it can aid in quick responses to cellular iron levels (Ma et al., 2012). There are still a lot of questions about how an active protein synthesis starting complex is put together.

**Figure 6:**
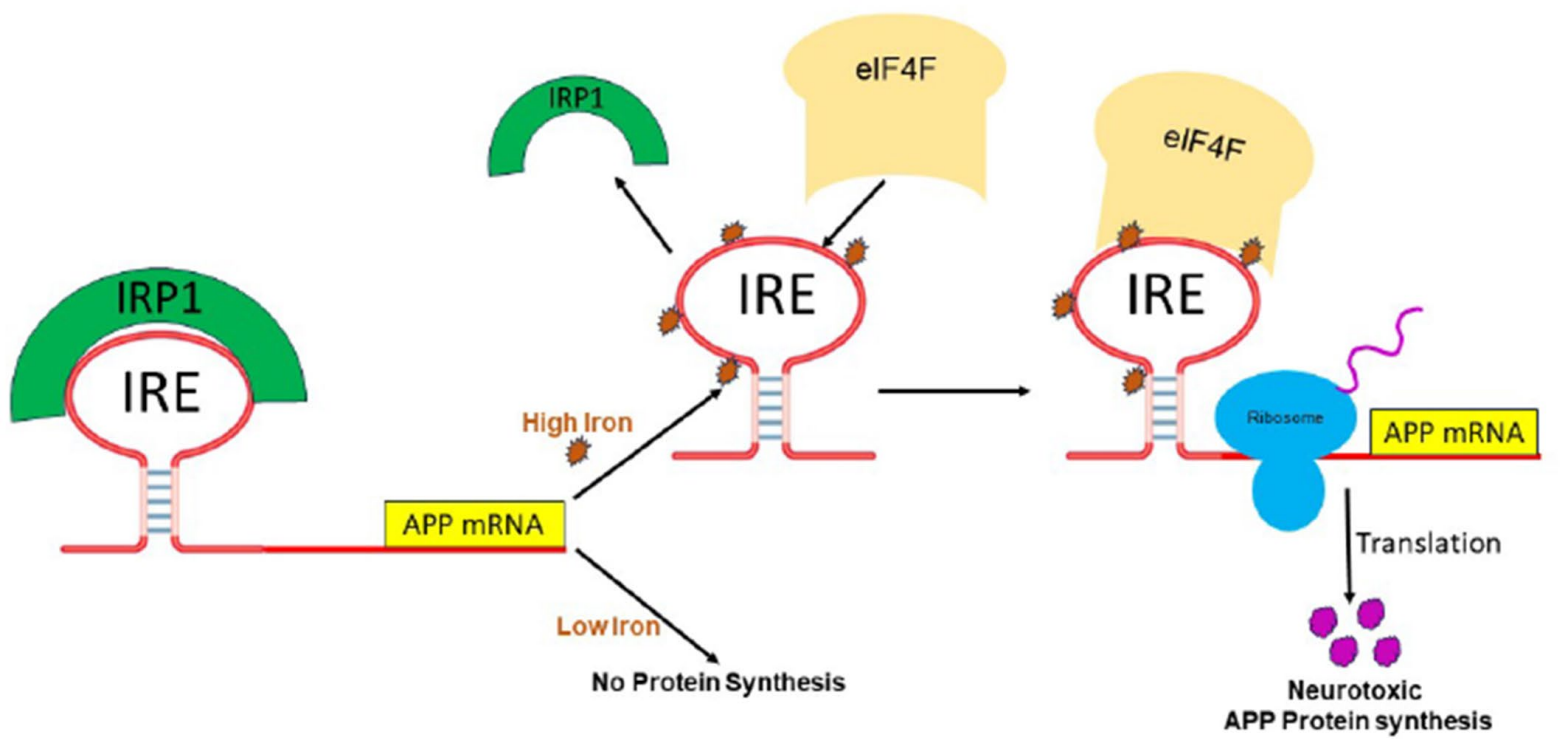
A possible mechanism of action for the molecular model explaining how elevated iron levels prevent IRP binding and encourage eIF4F and ribosome binding to start the translation of the neurotoxic protein by APP IRE RNA in the brain, hence contributing to Alzheimer’s disease.

We showed that IRE at the 5’-UTR of the target mRNA may selectively and strongly interact with translation initiation factor eIF4F, which is essential for translation initiation (Ma et al., 2012). On the other hand, iron can also bind to the IRE RNA directly, changing the structure of the mRNA. The interaction between IRE RNA and eIF4F, which can outcompete binding between IRE and IRP, will be facilitated by the structural changes in the mRNA caused by iron binding. Iron homeostasis will be hampered by disruption of the IRP/IRE signaling system, which may have a role in the initiation and progression of AD. The physiological iron signal on IRE RNA translation is represented by a proposed model (Figure 6) for iron-regulated neurotoxic amyloid protein production. For iron-induced protein biosynthesis to occur, IRE RNA must sequentially interact with IRP1, iron, eIFs, rRNA/protein complexes with ribosomes, and tRNA/protein elongation factor complexes. IRP1 binds significantly to the IRE RNA suppression of neurotoxic protein synthesis at low cellular iron levels, where ribosome binding and the initiation factor eIF4F are inhibited. On the other hand, iron can also directly attach to the target mRNA’s IRE at high cellular iron levels, changing the mRNA’s conformation. The detachment of IRPs from the target mRNA is encouraged by the structural changes in mRNA caused by iron binding. This facilitates the interaction between eIF4F and IRE RNA and speeds up the translation of the neurotoxic amyloid overproduction that causes AD (Figure 6).

## Iron homeostasis in brain

Iron is the most prevalent metal ion found in the central nervous system glial and neuronal cells. Since it is essential to brain function, maintaining neuronal iron homeostasis requires strict control of iron homeostasis (Long et al., 2022b; Long et al., 2022a; Ashraf et al., 2019; Alexiou et al., 2019). To ensure that the tissues of every organ in the body get the right amounts of iron, humans have evolved intricate processes. A series of interlocking mechanisms primarily control intracellular iron homeostasis in mammals, such as iron regulatory proteins and iron responsive elements mRNA, hepcidin-ferroportin-mediated serum iron level regulation, and hypoxia inducible factor-2α mediated transcription.

Proteins including ferritin, transferrin, and its receptors (TfR) control iron uptake, distribution, and sequestration inside cells (Wang and Wang, 2019). The relationship between astrocytes and endothelial cells controls the incorporation and transport of iron in the brain. There are no transferrin receptors on astrocytes. Ferric iron (Fe^3+^) loaded transferrin (Tf) is bound by TfR1 in the luminal membrane of endothelial cells, and this complex is internalized into the endosomal compartment where Fe^3+^ is converted to ferrous iron (Fe^2+^) (Rouault and Cooperman, 2006). A protein called TfR1 has a strong affinity for Fe^2+^ Tf. It combines with the TfR2 receptor to form a complex, which is then internalized into the endosomal compartment. Then, Fe^3+^ is reduced to Fe^2+^, enabling DMT1 to transport the complex across the endosomal membrane and into the cytosol (De Domenico et al., 2008; Ingrassia et al., 2019). Apo-Tf binds to the Tf receptor with great affinity in the acidic endosome. Before exocytosis, when the endosome combines with the lysosome, this contact stops the breakdown of free Tf. When the pH returns to neutral after exocytosis, the apo-Tf separates from the TfR1, thereby recycling the Tf molecule for additional usage in the circulation of iron. Because neurons express both DMT1 and TfR1, they can take up iron through a process known as receptor-mediated endocytosis (Rosenblum and Kosman, 2022; Belaidi and Bush, 2016).

Excess iron is stored in the cytoplasmic labile iron pool and mitochondria for the synthesis of iron–sulfur (Fe-S) clusters and heme prosthetic groups, to the cytoplasmic iron storage protein ferritin, and to the bloodstream by ferroportin (Fpn1) after it enters the cytoplasm of these cells and satisfies metabolic needs (Rouault and Cooperman, 2006). Ferroportin mediates the sole known export route in mammalian brain cells (Ganz, 2005). Ferroportin facilitates the export of ferrous iron from the cell; however, for Tf to bind the exported iron, ferroxidase must oxidize the ferrous iron to ferric. Both ferroportin and its related ferroxidases, amyloid precursor protein, have been found in brain astrocytes and neurons. The iron storage protein ferritin is made up of two subunits: the L-subunit oversees long-term iron storage, while the H-subunit has a ferroxidase activity that aids in the quick absorption and use of iron. Although H-ferritin is more widely distributed and the ratio of H-to-L-ferritin is dependent on the iron utilization by certain regional cells, both subunits are expressed in all regions of the brain (Madsen and Gitlin, 2007).

At birth, the brain has no iron. Between youth and middle adulthood, it rises quickly before staying mostly steady (Bartzokis et al., 2007). Glial cells higher quantities of iron are found in astrocytes, oligodendrocytes, and microglia than in neurons, making astrocytes the main iron-homeostatic cells in the central nervous system (Reinert et al., 2019). Since labile iron catalyzes the production of harmful reactive oxygen species, the minute cellular free iron pool is strictly controlled. Glial cells have ferritin, the primary protein that stores and buffers iron; neurons typically do not (Zecca et al., 2004). Iron regulatory proteins (IRPs), which bind RNA sequences known as iron responsive elements (IREs) in the 5′-untranslated region of ferritin mRNA, are primarily responsible for controlling ferritin production at the post-transcriptional stage (Walden et al., 2006). It has been demonstrated that under some circumstances, iron and other metals can accumulate in the choroid plexus and iron is regarded as a metal of intermediate toxicity with respect to its ability to harm the blood–brain barrier and the blood-cerebrospinal fluid barrier. Any disruption of homeostasis, such as genetic variations impacting metal excretion or increased exposure to iron in the environment (Calderón-Garcidueñas et al., 2020), may permit the build-up of iron, thereby making future neurodegeneration more likely. Patients with Alzheimer’s disease showed increased iron levels in the affected areas of their brains. A protein called ferritin controls and stores iron. Higher ferritin levels imply that preclinical AD is characterized by elevated iron levels in cerebrospinal fluid and plasma (Goozee et al., 2018). It has been suggested that iron deposits play a part in neurodegenerative illnesses. Humans have neurons in specific areas, such the substantia nigra (SN) and locus coeruleus (LC), that accumulate neuromelanin (NM), which subsequently sequesters excess labile iron (Dusek et al., 2022). It is interesting to note that SN is afflicted in AD, PD, LC, and these regions are frequently affected early in neurodegenerative illnesses (Fu et al., 2018; Ehrenberg et al., 2017).

Protein aggregates, a defining feature of neurodegenerative diseases, appear to draw iron and other metals, which function as glue and cause misfolding of proteins. There is a notable build-up of iron in and around amyloid senile plaques and neurofibrillary tangles (NFTs) in Alzheimer’s disease (Rajasekhar et al., 2015). This leads to changes in the way that iron regulatory proteins and IREs interact, as well as complications with iron sequestration and storage. Furthermore, the amyloid plaques in the Tg2576 mouse model for AD have been found to contain elevated levels of iron, which is similar to what is observed in AD patient’s brains (Ghosh et al., 2015). It is interesting to note that atypical iron responsive proteins are found in both amyloid precursor protein in Alzheimer’s disease and *α*-synuclein in Parkinson’s disease (Hofer and Perry, 2016; Ma et al., 2021). Therefore, α-synuclein and APP translation may be triggered by elevated cytosolic iron. Patients with AD had considerably higher serum iron levels (Sternberg et al., 2017). More research is still needed to determine the exact association between iron and the development of AD, but in any event, the body’s iron content must be strictly regulated. According to these investigations, iron particularly encourages the death of neural cells. AD’s neuronal loss is a key characteristic. Even though iron is a necessary metal for human health, excessive exposure to iron can impair normal nerve function and contribute to AD by causing neuronal death, oxidative stress, neuroinflammation, and disruption of neurotransmitter modulation. A number of neurodegenerative diseases appear to be influenced by disruptions in cerebral iron. Due to the iron buildup, neurons and glial cells degenerate, and there is neuroinflammation and immune cell infiltration at the locations of small lesions (Urrutia et al., 2021; Hinarejos et al., 2020). Increase in iron levels in Alzheimer’s disease brains correlated with ferritin in the surrounding neuroglia cells or with senile plaques and NFTs has previously been observed (Smith et al., 2007). Iron stimulates A*β* peptide aggregation and amyloidosis, and in cultured cells, iron complexed with Aβ peptides is harmful (Liu et al., 2011).

## Mis-regulation of brain iron in Alzheimer’s disease

Alzheimer’s disease pathogenesis is influenced by disruptions in the brain’s iron homeostasis (Fleming and Ponka, 2012; Chen and Olsthoorn, 2019). Report indicates that iron build-up and elevated iron concentrations (~1 mM) in the vicinity of amyloid plaques and NFTs in AD patients (Yin et al., 2021) have a substantial association with tau pathology and amyloid *β*-plaque pathology (Yin et al., 2021) as well as the pathogenesis of AD. Increased brain iron level directly linked with increased ferritin levels in plasma and CSF, which is a characteristic of preclinical AD (Goozee et al., 2018; Ayton et al., 2023). The development of Aβ plaques is accelerated by iron buildup (Pan et al., 2022; Ayton et al., 2020). According to reports, cellular iron levels control the translation of the APP (Bandyopadhyay and Rogers, 2014). Through IRE/IRPs, iron controls how APPs are processed (Bandyopadhyay and Rogers, 2014; Peng et al., 2021). It has been discovered that APOE4 increases the risk of AD via regulating ferritin, an iron homeostasis protein, and inversely regulating the effects of iron on brain function (Kagerer et al., 2020). The IRE has an impact on APP translation. By preserving the stability of FPN, APP can indirectly control neuronal iron levels and encourage iron export (Rogers et al., 2002). The FPN1-APP complex can be stabilized by tau proteins, which can also carry APP to the cell membrane (Belaidi et al., 2018).

**Figure 7:**
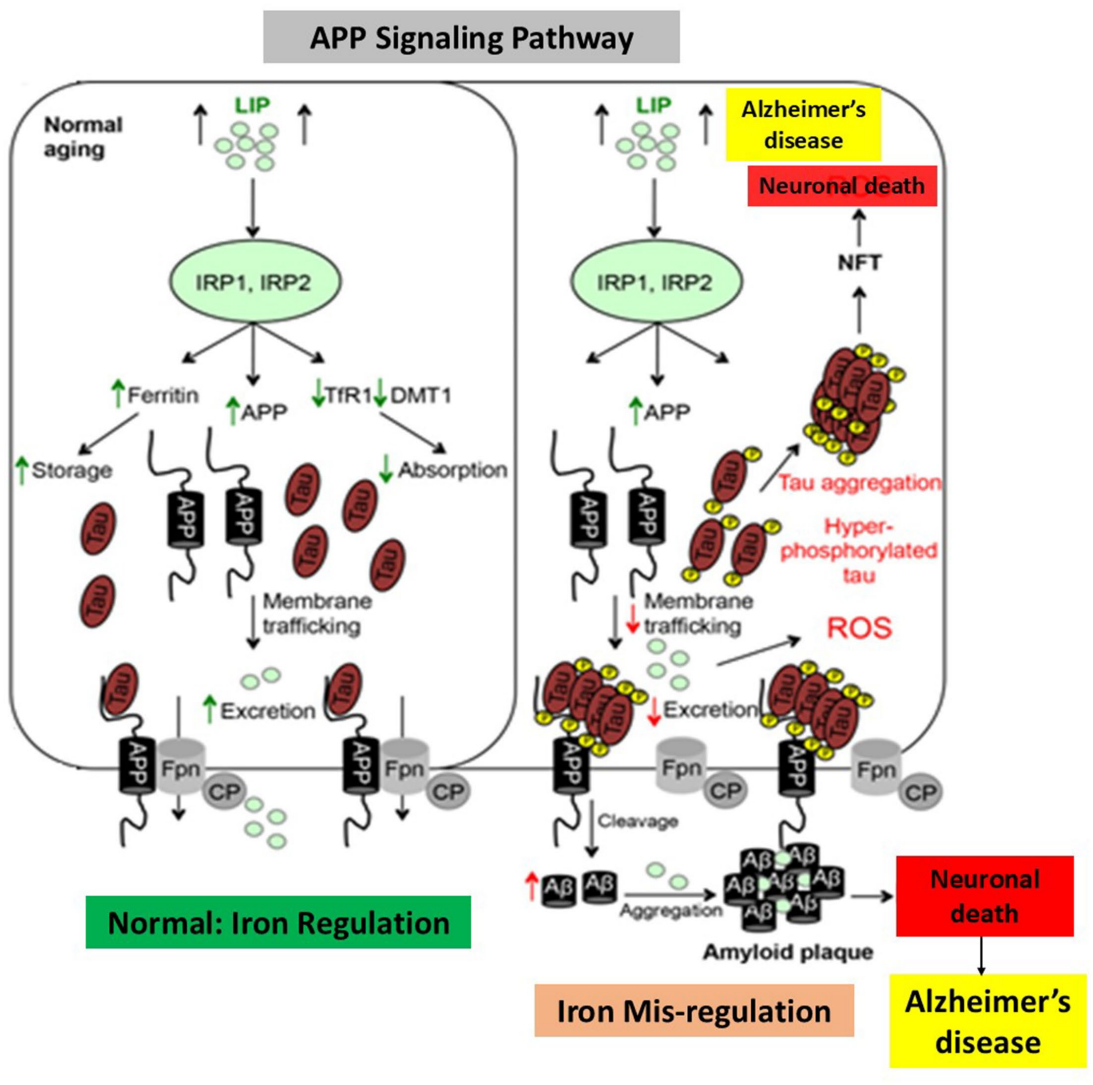
Alzheimer’s disease and dysregulation in brain iron homeostasis. A model that illustrates how alterations in reaction to elevated cellular iron levels in both healthy and Alzheimer’s disease patients cause iron-induced translation of amyloid plaque by APP IRE RNA. This picture, which depicts the sequences of events in normal individuals and Alzheimer’s disease in response to cellular iron, is modified from Belaidi and Bush (2016).

Additionally, excess iron encourages the production of tau protein hyperphosphorylation, while iron itself increases the creation of oligomeric tau (Wan et al., 2019). The process of brain iron mis regulation and its relationship to AD is illustrated in Figure 7, with a focus on the iron’s ability to bind to A*β*-peptide and increase amyloid-β toxicity. Hypothesized biochemical events in both AD and normal individuals in response to an increase in the labile iron pool of neurons, as seen in young, healthy individuals. Iron regulatory proteins are crucial for controlling the absorption, storage, and excretion of proteins by neuronal cells in healthy humans in response to an increase in the labile iron pool. Increased production of ferritin and amyloid precursor protein consequently leads to a rise in the labile iron pool, which promotes iron export and storage, respectively. On the other hand, decreased expression of transferrin receptor-1 and divalent metal transporter-1 inhibits iron import. By decreasing the amount of soluble tau (Figure 7), hyperphosphorylation and tau aggregation impede the transport of APP to the cell membrane, leading to an increase in labile iron buildup in neurons (Dusek et al., 2022; LeVine, 2024; LeVine et al., 2023). AD is also marked by increased APP cleavage into amyloid-β, which collects into amyloid plaques because to its strong affinity for iron, causing synapse loss and neuronal death.

Additionally, the buildup of iron in AD neurons causes other brain cells to become depleted of iron, which in turn causes neurodegeneration. The fact that cellular iron levels directly control APP translation through IREs found in the 5’UTR mRNA suggests a significant connection between iron homeostasis and Alzheimer’s disease (Rogers and Cahill, 2020). The transmembrane protein known as APP has been primarily investigated for its ability to cleave amyloid-β, although it also has a broad functional role in neural plasticity, synapse formation, and iron transport (Caldwell et al., 2013). *α*-secretase and *β*-secretase are involved in the non-amyloidogenic pathway, which is responsible for the proteolytic cleavage of the majority of neuronal APP under normal circumstances. The amyloidogenic process forms amyloid-β peptides by first cleaving APP with β-secretase and then *γ*-secretase. Increased iron concentrations in neurons increase the amount of APP expression that can undergo amyloidogenic processing and potentially enhance the generation of amyloid-β because iron levels regulate APP expression at the translational level. Through its stabilizing association with surface ferroprotein, APP has also been demonstrated to assist iron efflux from neurons; in cultured neurons and mice models, APP loss results in internal iron retention (Wan et al., 2012; McCarthy et al., 2014). Because tau deficiency inhibits APP trafficking to the membrane, it cannot interact with ferroportin and inhibits iron efflux from neurons, which results in intracellular iron accumulation and dopaminergic neuronal death, Parkinsonism and dementia. The presence of iron can influence how APP is processed; at high concentrations, iron prevents APP from maturing while leaving immature APP intact. A single substrate known as APP is the one that undergoes proteolysis to yield the 40–42 amino acid amyloid-beta peptide, which then combines to form the primary building block of amyloid plaques (Cahill et al., 2009). Translational expression of immature APP results in increased neurotoxic oxidative stress and impaired iron metabolism, both of which contribute to Fe^2+^-induced neurotoxicity. The majority of neuronal APP is normally cleaved by proteases, and the big N-terminal fragment sAPPα, which possesses characteristics that support cell development and protection, is released by α-secretase and γ-secretase that belong to the non-amyloidogenic route (Müller et al., 2017). Tau deficiency prevents APP from moving to the membrane, which prevents it from interacting with Fpn and prevents neurons from releasing iron. This leads to intracellular iron buildup, dopaminergic neuronal death, Alzheimer’s disease, dementia, and Parkinsonism.

Undoubtedly, one of the ultrastructural conditions required for the polymerization of Aβ peptide is iron (Lei et al., 2021). One clear connection between high iron levels and the known loss of neuronal function observed in AD patients is that iron accelerated the activity of β-amyloid to accelerate Aβ-induced neuroblastoma cell death. The expression of multiple iron proteins is altered in AD brains, indicating an iron imbalance. One iron protein that is highly expressed in AD patients is iron store ferritin. In the AD brain, excess iron buildup is consistently observed. Elevated levels of iron and ferritin-rich cells have been found in brain postmortem samples from amyloid plaques in AD patients (Zecca et al., 2004). Iron chelation lowers the synthesis of APP (Bandyopadhyay et al., 2010). The pathophysiology of Alzheimer’s disease is linked to iron trafficking and buildup (Hin et al., 2021). From a mechanical standpoint, iron acts as a pathogenic regulator of APP translation and amyloid toxicity. An essential connection between iron homeostasis and AD is the regulation of amyloid precursor protein expression by cellular iron levels via IREs found at the 5’-UTR mRNA (Rogers et al., 2002). The 146 nt 5’-UTR of APP mRNA (+51 to +94 from the 5’-cap site) contains a distinct CAGA box, amyloid, which binds to the IRE binding protein IRP1 and is encoded by the APP mRNA. By modifying mRNA translation and stability of mRNAs for the iron-associated proteins, APP, ferritin, and transferrin receptor, IRPs are adequate to control intracellular iron homeostasis (Bandyopadhyay et al., 2013). Therefore, alterations in the amounts of iron within cells have the potential to disrupt the binding of IRP1 to IRE RNA stem loops, impact transferrin-ferritin balance (Wang and Pantopoulos, 2012), and influence the iron export protein APP. These findings support the roles of the transferrin and hemochromatosis genes as hereditary variables that may raise the incidence of late-onset sporadic AD. When combined, iron can influence the development of Alzheimer’s disease by controlling the levels of APP, amyloid-*β*, and tau protein hyperphosphorylation. Thus, the present emphasis of RNA-based anti-amyloid therapy is on the IRE RNA stem loop in APP RNA. Future research is needed to examine how iron and APP relate to the pathophysiology of AD.

A number of gene loci, including APP, presenilin genes, PS1, and PS2, have been found by genome-wide association studies (GWAS) to affect the risk of AD. The expression of proteins implicated in the amyloid-beta breakdown of AD pathogenesis is known to be influenced by the products of several gene loci. Through controlling the expression and functionality of amyloid proteins, recent research (Olufunmilayo and Holsinger, 2023) has offered compelling evidence linking non-coding RNAs to AD. The pathophysiology of AD is linked to iron and the IRP/IRE signaling system. More than 20 AD risk loci have been found since the development of next-generation sequencing and GWAS (Karch and Goate, 2015). Apolipoprotein E (APOE), the most potent risk factor for sporadic AD, was discovered far earlier than high-throughput sequencing. Additionally, the active site for the microRNA (miR-346), which has been shown to increase APP translation, was discovered to overlap with the IRE of APP mRNA in the 5’-UTR (Long et al., 2019). MiR-346 has been shown to target the APP mRNA 5’-UTR, resulting in an overexpression of APP that may have implications for iron homeostasis in AD (Long et al., 2019). With implications for its function when iron interfaces with brain levels of APP and amyloidosis in AD, this work detailed how iron regulatory protein (IRP1) and miR-346 interact to bind and modify the activity of the APP IRE mRNA (Cho et al., 2010).

## Conclusions and future perspectives

There is no doubt that APP, iron, IRP, and IRE RNA are closely related and that each of these effects Alzheimer’s disease are different. Research on APP IRE RNA and IRP in brain iron metabolism disorders has undoubtedly increased the relevance of these findings for AD pathogenesis and treatment approaches. According to the current study, brain iron overload, amyloid-β metabolic disorders, tau protein hyperphosphorylation, oxidative stress and free radical damage, cholinergic neuron loss, inflammatory damage, and gene mutations are potential pathophysiological factors associated with Alzheimer’s disease. The many mechanisms involved in AD development make it difficult to create treatments for the disease.

Alzheimer’s disease is characterized by dysregulation of the brain’s iron levels and the proteins that bind them. The ramifications of our finding of completely functional IRE RNA stem loops in the 5’-UTRs of the AD-specific APP RNA, which encode important proteins and the agents responsible for neurodegenerative disorders, are a crucial part of this review. Selective RNA targeting of APP translation through the uniquely folded 5′-untranslated region of the precursor transcript can suppress APP expression (Bandyopadhyay et al., 2013), providing a novel target to supplement current tactics. A method to find and describe inhibitors of this process involves applying knowledge of the translational control circuits by which iron controls translation of the iron storage proteins ferritin and APP through interactions with their respective 5’-UTR, each of which encodes distinct versions of IRE (Cho et al., 2010). While 3-D structure is a more typical pharmacological target for proteins, RNA secondary structure plays a major role in the current development of RNA medicines, such as those based on RNAi. Since RNA targets are smaller than protein targets, RNA therapeutics have an advantage over protein therapies. Small compounds can change the function of mRNA in solution and in cultured human cells by binding to specific locations in the IRE RNA (Yang et al., 2023; Childs-Disney et al., 2022). These findings demonstrated that the small RNA binding molecules had the same selectivity in binding to folded target RNA structures in solution as they do when they enter living cells. Target regions of the IRE RNA will be identified for upcoming drug design studies as part of ongoing research to characterize the iron binding site on the IRE. Animal IRE RNAs have evolved during evolutionary time and serve as a model system for other 3-D mRNAs in all organisms. Proof of principle data about small molecules targeting mRNA structure has been obtained by IRE RNAs, demonstrating the unexplored possibility of chemical manipulation of mRNA and protein synthesis in live systems. In certain situations, such as IRP-inactivated ferritin mRNA, a little molecule may be able to activate the portion of mRNA that is repressed in iron overload and prevent iron chelators from causing damaged ferritin and iron overload to build up. Another example involves the potential therapeutic utility of RNA-based small compounds as APP translation inhibitors that target the mRNAs associated with Alzheimer’s disease.
